# Evaluation of ambulatory electrocardiographic monitoring of patients after high-risk acute coronary syndrome: the MONITOR ACS-Epic 13 randomized trial

**DOI:** 10.3389/fcvm.2025.1646175

**Published:** 2025-08-18

**Authors:** Jose M. De La Torre Hernandez, Victor Exposito, Susana Gonzalez Enriquez, Felipe Rodriguez Entem, Adrian Margarida, Tamara García Camarero, Gabriela Veiga, Fermin Sainz Laso, Dae-Hyun Lee, Aritz Gil Ongay, Sergio Barrera, Santiago Catoya, Celia Garilleti, Rigoberto Hernandez, Cristina Obregon, Juan J. Olalla

**Affiliations:** Cardiology Division, Hospital Universitario Marques de Valdecilla, IDIVAL, Santander, Spain

**Keywords:** atrial fibrillation, cardiac arrhythmias, myocardial infarction, continuous monitoring, implantable loop recorder, wearable device

## Abstract

**Aims:**

Patients with acute coronary syndrome (ACS) may experience adverse events during follow-up. Previous trials have shown that asymptomatic arrhythmias preceded these adverse events in a substantial proportion of patients. Ambulatory remote monitoring may allow early detection of electrocardiographic alterations with therapeutic and prognostic implications. This study aims to evaluate the efficacy of remote monitoring in this population.

**Methods and results:**

This is a single-center, randomized controlled trial designed to evaluate the efficacy of 12-month monitoring of high-risk patients after ACS. High risk was defined by a GRACE score over 118 and a CHA_2_DS_2_-VASc score over 2. In the intervention arm, two different systems were implemented: an implantable loop recorder in all patients and a portable mobile ECG system in those suitable. The primary endpoint was detection rates for atrial fibrillation/flutter, ventricular arrhythmias, and advanced conduction abnormalities. A total of 150 patients (estimated sample size) were included. Baseline clinical characteristics were comparable. The primary endpoint was achieved in 21.3% of the patients in the monitoring group and only in 2.7% in the control group (*p* = 0.0005). The combined diagnosis of atrial fibrillation/flutter was significantly more frequent in the monitoring group (10.6% vs. 1.3%, *p* = 0.016). The incidence of death, myocardial infarction, or stroke at 12 months was 9.3% in the control group and 5.3% in the monitoring group (*p* = 0.34).

**Conclusions:**

Ambulatory monitoring of high-risk patients after ACS shows a significantly higher detection of ECG-clinically relevant findings as compared with a control group. This was not translated into a difference in 1-year MACE, though the study was clearly underpowered for these clinical endpoints.

**Clinical Trial Registration:**

https://clinicaltrials.gov/study/NCT03940066?tab=history&a=3NCT03940066, identifier: NCT03940066.

## Graphical Abstract

**Figure:**
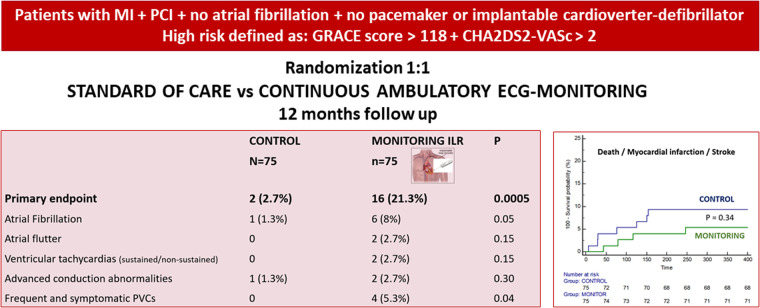

## Introduction

Patients with acute ST or non-ST-elevated myocardial infarction (STEMI/NSTEMI) may suffer a high incidence of adverse events in short- to medium-term follow-up (1). Some of these events have an electrocardiographic expression (mainly alterations in cardiac rhythm, along with other potential findings such as ST changes) and may have a potential relationship with serious clinical outcomes such as death, infarction, or stroke, as well as readmissions. Recent data from observational studies have shown that incident (asymptomatic) arrhythmias in post-MI patients are more predictive for MACE than a large number of known demographic, clinical, and diagnostic risk parameters (2–4).

Improved efforts to detect these rhythm disturbances in this setting are warranted, as they may lead to therapeutic actions that, if implemented in time, may improve prognosis. However, it can be challenging given the paroxysmal and asymptomatic course of most arrhythmia episodes. These limitations may be overcome by implantable loop recorders (ILR) in a meaningful proportion of patients after myocardial infarction.

Although data on the real incidence of post-MI arrhythmias are very limited, a growing body of evidence indicates that atrial fibrillation (AF) and non-sustained and sustained ventricular tachycardia (NSVT, VT) likely account for a proportion of these events. The reported prevalence of new AF after acute coronary syndrome (ACS) is variably reported, ranging from 6% to 21% (5, 6), and even 58% in the mid-term follow-up in recent trials using ILR improved detection algorithms, with a 30%–40% occurring late (after discharge) (4, 7). Importantly, most patients remain unaware of these AF episodes, although late detection of AF in this population further worsens prognosis at 1-year follow-up than early AF (8). On the other hand, device-detected subclinical AF is associated with an increased risk of ischemic stroke (9). The incidence of NSVT in patients following a myocardial infarction has been previously determined mainly by 24 h Holter monitoring. Its occurrence has predicted both non-sudden and sudden cardiac death in investigations conducted in the present therapeutic era (10). However, data regarding its prevalence are lacking, with data disparity due to different diagnostic criteria.

The objective of this trial is to assess the usefulness and efficacy of remote continuous monitoring for patients at high risk of adverse events after an acute coronary syndrome.

Secondarily, two different systems are compared within the intervention arm: an implantable loop recorder (ILR) and a portable, mobile, connected ECG device (PM-ECG).

## Methods

### Study population

This is a single-center, randomized controlled trial designed to evaluate the efficacy of 12-month post-discharge monitoring of patients with high-risk MI defined by GRACE and CHA2DS2-VASC scores. Although CHA2DS2-VASC was designed to estimate the risk of embolic events in patients with already known AF, there is a strong association between increasing score and risk of new-onset arrhythmias and MACEs (3, 11–16).

The cutoff points in our study were established after a preliminary study in a contemporary cohort of 327 patients undergoing PCI after STEMI/NSTEMI in our center, establishing a difference in MACEs at 12 months of 20% vs. 7% in 108 patients with GRACE >118 and CHA_2_DS_2_-VASs >2 and 145 patients with lower values for both.

In the intervention arm, two different systems were implemented: an ILR in all patients and a PM-ECG in those suitable.

Inclusion criteria: (1) patients over 18 years old who can understand the nature of the study and have provided written informed consent; (2) diagnosis of ACS, either STEMI or NSTEMI; (3) percutaneous revascularization of significant culprit lesions; (4) GRACE score 6-month mortality over 118; and (5) CHA_2_DS_2_-VASc score over 2.

Exclusion criteria: (1) history of AF; (2) episodes of AF during admission; (3) pacemaker or ICD previously implanted or under current indication; (4) participation in another clinical trial at the time of inclusion in the study; (5) life expectancy <2 years; (6) pregnant or breastfeeding women; (7) unable to provide consent for participation.

After information and signed consent, patients were randomized to the control group and the monitoring group.

### Study procedures

In the monitoring group, an ILR (BioMonitor 2, Biotronik SE & Co., Germany) was implanted in all patients before discharge, and the feasibility of using the PM-ECG (KardiaMobile, AliveCor, United States) was assessed, in which case the use of the device was educated and facilitated.

In all patients with an ILR implanted, automatic monitor-sensed or symptom-triggered ILR recordings were collected. The device used the 6 min (nominal) range to tag an AF episode using R–R interval variation algorithms. Once it identifies it, it sends up to six ECG strips of 60 s for confirmation by the physician reviewing the episode. To define VT, we used nominal settings with a cycle length of 333 ms (180 bpm for ≥16 beats).

In the subgroup of patients using PM-ECG, routine e-mailed or symptom-triggered records were collected.

The ILR/PM-ECG records were reviewed by two electrophysiologists with extensive experience in monitoring and ECG interpretation, and in case of discrepancy between them, a third one was consulted to label the episode.

**Figure 1:**
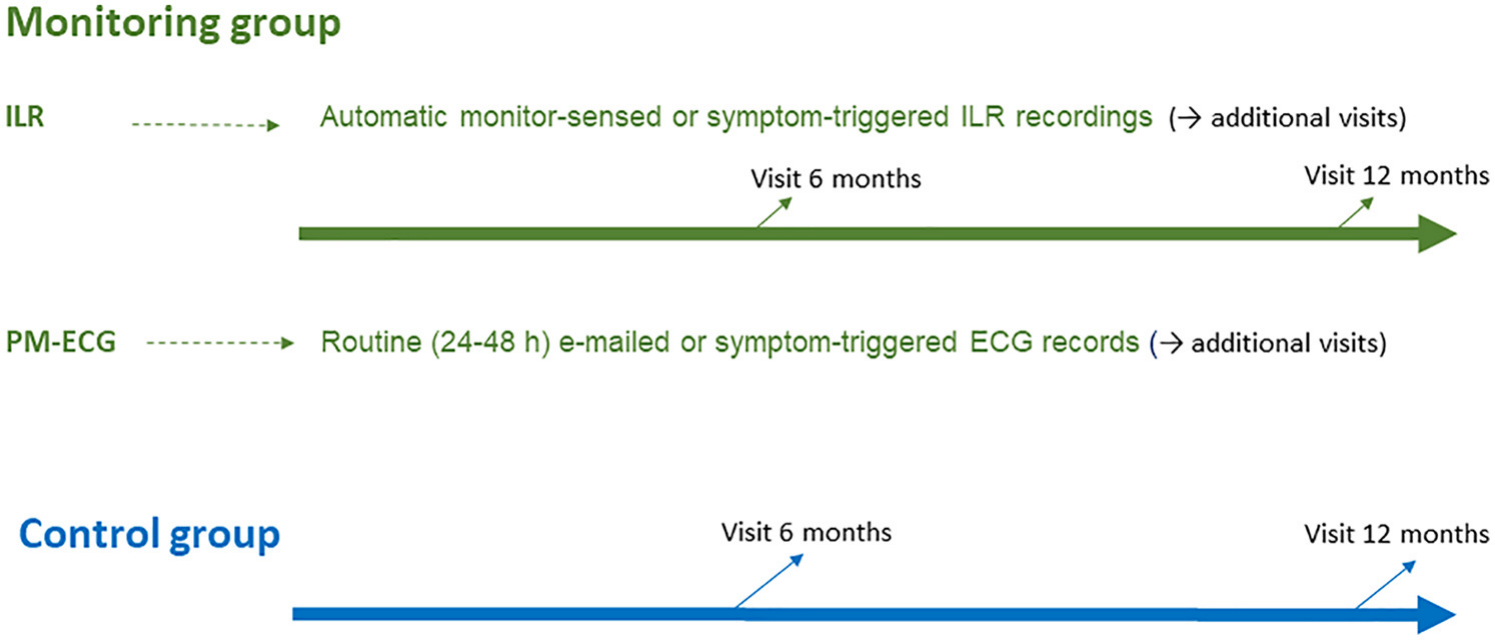
Schematic of the follow-up plan in the trial for both arms.

On-site visits at 6 and 12 months after discharge were scheduled in both groups. In the monitoring group, any extra visits could be indicated according to findings (Figure 1).

The study was approved by the corresponding Institutional Review Board and was registered (NCT03940066). All participating patients signed the informed consent after a proper explanation of the investigational procedures. The database was completely anonymized.

### Endpoints and classification of findings in the monitoring group

The primary endpoint of the trial was the detection rate for the composite of significant arrhythmia detection (AF/flutter episodes of significant duration, other sustained supraventricular arrhythmias, sustained or non-sustained ventricular arrhythmias, advanced heart conduction abnormalities).

The secondary endpoints were as follows: (1) incidence of major adverse cardiovascular events [death, myocardial infarction, and stroke (MACE)]; (2) diagnosis of AF or AFL inducing therapeutic actions; and (3) correlation of ILR vs. PM-ECG for detection of electrocardiographic events.

The electrocardiographic findings in the monitoring group were classified as clinically relevant when AF or AFL last >6 min (17); symptomatic, sustained or recurrent supraventricular tachycardias; ventricular tachycardias (non-sustained or sustained); sinus pauses longer than >3.5 s, symptomatic, diurnal, or out of a vagal context; type 2 second-degree AV block or third-degree AV block; and frequent and symptomatic ventricular premature beats (VPB).

### Statistical analysis

According to a superiority design for a binary outcome, a sample of 146 patients (73 per group) was estimated to have an 80% probability of detecting a significant (*p* < 0.05) increase in the primary endpoint, estimated 5%–10% in the control group and at least 20%–25% in the monitoring group. The value of 5%–10% in the control group comes from data from our experience and from registries (5, 6, 8). Although the incidence of adverse events is expected to be high in the group of patients to be included, the detection of potentially precursor electrocardiographic alterations in the scheduled outpatient visits could be somewhat lower. The value of 20%–25% in the monitoring group comes from a conservative estimate derived from studies using ambulatory monitoring in patients after acute coronary syndrome, and considering our criteria for significant electrocardiographic findings (4, 7).

Continuous variables are presented as mean ± standard deviation or median (interquartile range) according to type of distribution, and categorical variables as percentages. Distribution was assessed for each variable with the Shapiro–Wilk test. Accordingly, continuous variables were compared with the Student's *t*-test if they followed a normal distribution and by non-parametric tests when this was not the case. The categorical variables were compared with the chi-square test or Fisher exact test, as required.

Kaplan–Meier curves for event-free survival were obtained for each group and compared using the log-rank test and the hazard ratios with 95% confidence interval. Multivariable logistic regression analysis identified independent predictors of the primary endpoint. Covariates that showed a univariate relationship with outcome (*p* < 0.2) were entered into the multivariable logistic regression model. Then, stepwise elimination analysis was performed to define a useful subset of predictors. Values of *p* < 0.05 were considered statistically significant. The statistical packages SPSS 25.0 and Medcalc 20.009 were used throughout.

## Results

### Study population

**Figure 2:**
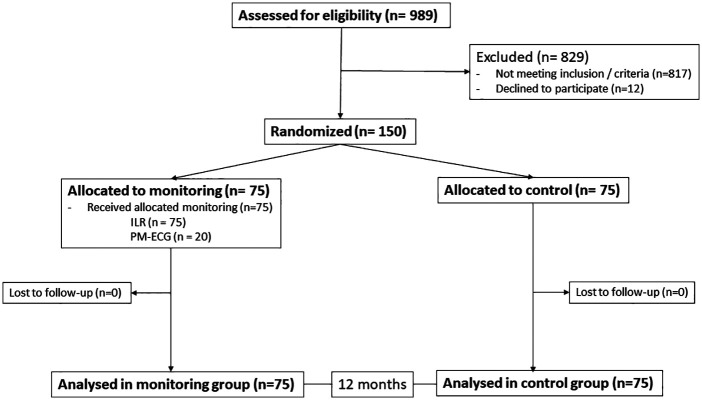
Flowchart of the study.

A total of 150 patients were included in the study. The chart of the trial is shown in Figure 2. Seventy-five patients were randomized to each group, and within the monitoring group, all 75 received the ILR, and a subgroup of 20 accepted and were adequately managed with the PM-ECG.

The baseline clinical characteristics of the patients were comparable, with nearly a third being women, a mean age of approximately 70 years, a higher proportion of STEMI than NSTEMI, similar values for risk scores, laboratory values, and echocardiographic and electrocardiographic parameters (Table 1). At discharge, comparable values were again observed for the most relevant analytical parameters and electrocardiographic and echocardiographic measurements. As for the revascularization procedures, the results were comparable in terms of technical aspects, number of vessels treated, total stent length, use of intravenous antiplatelet agents, and angiographic results (Table 2).

**Table 1:** Clinical characteristics.

Variables	Control	Monitoring	*P*
	*N* = 75	*n* = 75	
Age, years	70.9 ± 8	69.8 ± 8	0.39
Women	24 (32%)	23 (30.7%)	0.86
Family history of CAD	9 (12%)	11 (14.7%)	0.62
Smoker	15 (20%)	15 (20%)	1
Diabetes	21 (28%)	24 (32%)	0.59
Hypertension	59 (78.7%)	52 (69.3%)	0.21
Dyslipidemia	43 (57.3%)	46 (61.3%)	0.62
Previous MI	11 (14.7%)	11 (14.7%)	1
Previous PCI	13 (17.3%)	10 (13.3%)	0.49
Previous CABG	0	0	1
Previous stroke	5 (6.6%)	4 (5.3%)	0.73
STEMI	46 (61.3%)	44 (58.7%)	0.70
NSTEMI	29 (38.7%)	31 (41.3%)	0.77
CHA_2_DS_2_-VASc score	3.2 ± 0.65	3.3 ± 0.7	0.36
GRACE score	130.5 ± 13.4	127.7 ± 10	0.14
PRECISE DAPT	17.5 ± 8.3	16.4 ± 8.1	0.41
LVEF,%	47 ± 9	46 ± 9.7	0.51
MR moderate/severe	2/0 (2.7%)	2/0 (2.7%)	1
Hemoglobin, g/dl	13.4 ± 8.3	13.7 ± 1.5	0.34
GFR, ml/min	73 ± 20	74 ± 17	0.74
PR interval, ms	164 ± 37	158 ± 32	0.28
QRS duration, ms	102 ± 18	103 ± 12	0.68
Bundle branch block L/R	1/8 (12%)	1/5 (8%)	0.41
*At discharge*			
LVEF,%	48 ± 10	46.4 ± 10	0.32
MR moderate/severe	1/0 (1.3%)	3/0 (4%)	0.30
Hemoglobin, g/dl	14.5 ± 1.5	14 ± 1.9	0.07
GFR, ml/min	73.7 ± 19	74 ± 16.5	0.91
PR interval, ms	151 ± 29	146 ± 21	0.22
QRS duration, ms	104 ± 12	103 ± 9	0.56
Bundle branch block L/R	0/8 (10.6%)	0/5 (6.7%)	0.37

Data presented as mean ± SD or *n* (%).

CABG, coronary artery bypass graft; CAD, coronary artery disease; GFR, glomerular filtration rate; LVEF, left ventricular ejection fraction; MI, myocardial infarction; MR, mitral regurgitation; PCI, percutaneous coronary intervention; STEMI, ST-elevated myocardial infarction; NSTEMI, non-ST-elevated myocardial infarction.

**Table 2:** Procedural characteristics.

Variables	Control	Monitoring	*P*
	*N* = 75	*n* = 75	
Radial	65 (86.6%)	67 (89.3%)	0.57
Primary PCI	46 (61.3%)	44 (58.7%)	0.70
PCI in three vessels	11 (14.7%)	8 (10.7%)	0.46
PCI in two vessels	22 (29.3%)	18 (24%)	0.41
PCI in one vessel	42 (56%)	49 (65.3%)	0.26
Total stent length, mm	44 ± 27	40 ± 26	0.35
Stent diameter, mm	3 ± 0.45	3.08 ± 0.55	0.33
IIb–IIIa inhibitors	6 (8)	4 (5.3)	0.50
Angiographic success^a^	73 (97.3%)	72 (96%)	0.66
DAPT for 12 months	73 (97.3%)	74 (98.7%)	0.69
Prasugrel or ticagrelor	49 (65.3%)	45 (60%)	0.52
Oral anticoagulation	0	2 (2.7)	0.15

Data presented as mean ± SD or *n* (%).

DAPT, dual antiplatelet therapy; PCI, percutaneous coronary intervention.

^a^ Angiographic success defined as residual stenosis <30% and TIMI III flow.

### End points and findings by monitoring

After 1 year of follow-up, the primary end point was achieved in 21.3% of the patients in the monitoring group and only in 2.7% of the patients in the control group (*p* = 0.0.0005, Table 3).

**Table 3:** Primary endpoint and its individual components.

Endpoints	Control	Monitoring ILR	*P*
	*N* = 75	*n* = 75	
Primary endpoint	2 (2.7%)	16 (21.3%)	0.0005
Atrial fibrillation	1 (1.3%)	6 (8%)	0.05
Atrial flutter	0	2 (2.7%)	0.15
Ventricular tachycardias	0	2 (2.7%)	0.15
Advanced conduction blocks	1 (1.3%)	2 (2.7%)	0.30
Frequent and symptomatic PVCs	0	4 (5.3%)	0.04

ILR, implantable loop recorder; PVCs, premature ventricular contractions.

The findings in the group with ambulatory monitoring are shown in Supplementary Table S1. The cumulative detection rate of clinically relevant findings was 16% after 6 months (12 patients) and 21.3% at the end of the study period (16 patients). Slightly more than half of these events were asymptomatic, or at least did not induce activation of the recording by the patient, and were obtained by automatic sensing. In the control group, two patients experienced relevant arrhythmias, with detection of asymptomatic AF in one case, and asymptomatic bradyarrhythmia in another one.

The most frequent type of finding was VPB, followed by supraventricular tachycardias, with AF of significant duration detected in six (8%) patients and AFL in two patients. Of the patients with detection of AF, two patients had atrial episodes <1 h long, two patients had episodes 12 h or longer, and two developed persistent AF. The median time to AF detection was 146 days (range, 47–350), with 5/6 detected in the first 6 months of follow-up. AFL was detected at 32 and 68 days after MI.

ILR activation by the patients in the presence of symptoms occurred in 41 patients (54.6%). In the great majority of these activations, no alteration was detected (70.5%); the most frequent finding was VPB, and only 1.6% of the activated recordings corresponded to AF. Two patient activations were NSVT, one accompanying chest pain and ST segment elevation in the context of vasospastic angina and another one because of palpitations (see Supplementary Figure S1).

Monitoring findings resulted in treatment changes in 13 patients, which represents 17.3% of all patients in the monitoring group and 81.2% of those with findings. These changes included three interventional procedures (AFL ablation in two patients and pacemaker implantation in another one) and pharmacological therapeutic changes, including the initiation of oral anticoagulation in five patients, calcioantagonists (*n* = 1) or beta-blockers (*n* = 4), plus discontinuation of negative chronotropic drugs in one patient with severe bradycardia (Supplementary Figure S2).

In the subgroup of 20 patients with PM-ECG, the device detected one asymptomatic AFL in a routine registry and two cases of frequent VPB in symptom-based registries. On the other hand, in this same subgroup, three relevant events (one case of second-degree AV block, one case of AF, and one case of NSVT) were non-detected by the PM-ECG, but automatically detected by the ILR (not triggered by symptoms).

The analysis of predictors for the primary end point identified dyslipidemia (HR 0.13, 95% CI 0.029–0.61; *p* = 0.01) and smoking (HR 3.77, 95% CI 1.01–14.42; *p* = 0.04) as independent predictors (Supplementary Table S2).

### Major adverse cardiac and cerebrovascular events

**Figure 3:**
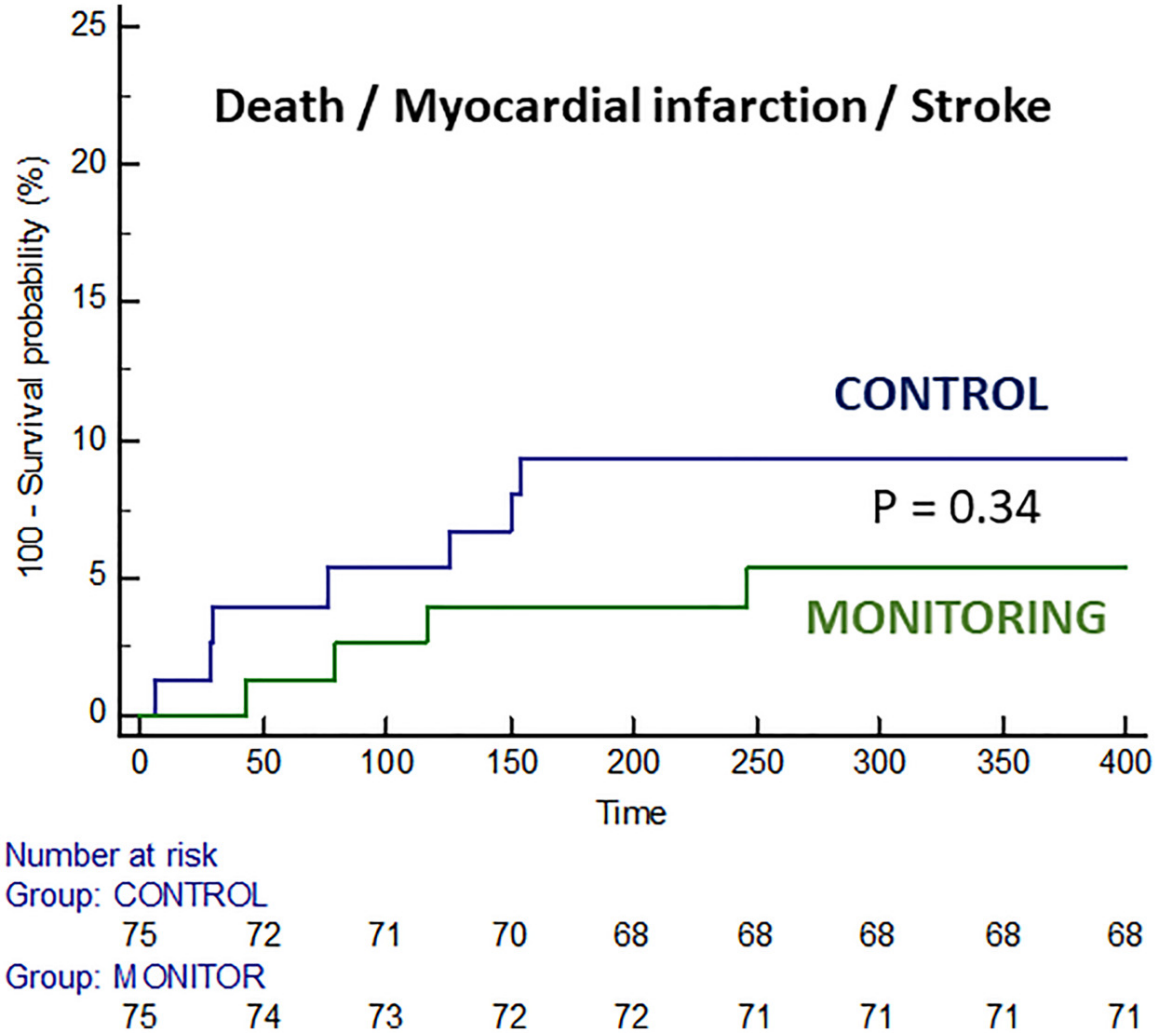
Survival curves free of death, myocardial infarction, and stroke in both groups.

Clinical events during follow-up are summarized in Table 4. There was no significant difference in the rates of major adverse cardiac and cerebrovascular events. The incidence of death, MI, or stroke was 9.3% in the control group and 5.3% in the monitoring group (*p* = 0.34), although the trial was underpowered for these major adverse cardiovascular events (Figure 3). The combined diagnosis of AF + AFL with therapeutic implications was significantly more frequent in the monitoring group (10.6% vs. 1.3%, *p* = 0.016).

**Table 4:** Clinical outcomes at 12 months.

Outcomes	Control	Monitoring	*p*
	*N* = 75	*n* = 75	
Death	3 (4%)	1 (1.3%)	0.30
Myocardial infarction	3 (4%)	4 (5.3%)	0.46
Stroke	1 (1.3%)	0	0.32
Death/MI/stroke	7 (9.3%)	4 (5.3%)	0.34
Bleeding BARC ≥2	5 (6.7%)	1 (1.3%)	0.09
Revascularization	2 (2.7%)	3 (4%)	0.65
Diagnosis of AFib/AFlut	1 + 0 (1.3%)	6 + 2 (10.6%)	0.016
Advanced heart blocks	1 (1.3%)	2 (2.7%)	0.30
Ventricular tachycardias	0	2 (2.7%)	0.08

AFib, atrial fibrillation; AFlut, atrial flutter; BARC, bleeding academic research consortium; MI, myocardial infarction.

The most relevant findings of the trial are illustrated in the “Graphical abstract.”

## Discussion

The main finding of our study is that systematic 12-month ambulatory monitoring using ILR in high-risk patients after ACS significantly increased the detection rate of clinically relevant arrhythmias, predominantly AF and AFL, as well as other electrocardiographic abnormalities compared with standard follow-up. Specifically, the monitoring group had a detection rate of 21.3% for clinically significant findings vs. 2.7% in the control group. This is consistent with prior reports indicating that a substantial proportion of post-ACS patients may experience previously unknown arrhythmias. Importantly, most of these episodes were asymptomatic and detected by automatic sensing, which highlights the limitations of symptom-based monitoring approaches in accurately identifying arrhythmias that may require intervention. Extended rhythm monitoring is particularly effective in identifying silent arrhythmias that can increase the likelihood of adverse outcomes. Cardiac arrhythmias were the most powerful predictor for MACE in several observational and randomized studies using ILR (3, 4, 18).

The identification of AF in the monitoring group is particularly noteworthy, as the presence of subclinical AF is known to be associated with an increased risk of ischemic stroke and other thromboembolic complications, heart failure, and death (19, 20). The combined incidence of AF and AFL in our study was slightly higher than reported at 12 months (21). The selection of a high-risk group of patients and the use of modern ILR with improved detection algorithms may explain these numbers. It is also noteworthy that in our series was a high burden of AF, including two patients who developed persistent AF. Our study confirmed that the majority of AF episodes occurred within the first 6 months following MI, suggesting a potential window of opportunity for early therapeutic intervention. In our series, these monitoring-derived findings led to a change in therapeutic management, including initiation of oral anticoagulation in five patients and interventional procedures such as AFL ablation and pacemaker implantation in three others.

Interestingly, no sustained VT were found during the continuous monitoring. Our incidence of ventricular tachycardia was similar to SMART-MI-DZHK9 and numerically lower than in previously published CARISMA, as we followed more stringent non-sustained ventricular tachycardia criteria adopted from the modern implantable cardioverter defibrillator programming era.

Another key finding was the ability of the ILR to detect a broader range of arrhythmias and conduction disturbances compared with the PM-ECG, which was used in a subgroup of patients. While the PM-ECG provided valuable information, the ILR proved superior in identifying significant events such as advanced atrioventricular block and sustained ventricular tachycardia, which were not captured by the portable device. This suggests that while mobile ECG devices are useful for symptom-triggered recordings, their diagnostic yield may be limited compared with continuous monitoring systems, which may be critical for detecting high-risk arrhythmias even in asymptomatic patients (22).

Predictive analysis identified smoking status as a significant independent predictor for the primary endpoint, which aligns with the well-established proarrhythmic effects of smoking (23). Interestingly, dyslipidaemia emerged as a protective factor, which we speculate may be related to more aggressive statin therapy in this subgroup, though this remains to be confirmed in future studies (24).

Whether clinically relevant events can be prevented more effectively through this early detection via continuous monitoring remains unanswered, as our study failed to demonstrate improved outcomes with this strategy in post-MI patients with mildly reduced or preserved ejection fraction. This lack of difference in hard clinical outcomes is likely due to limited sample size and low event rates. The recently published BIOGUARD-MI trial also failed to show an improved outcome in post-infarction patients without ICD indication and remote monitoring by ILR (25). Although it enrolled a substantially larger population with a high-risk selection criteria slightly different, the unbiased collection of adverse events and the lack of regular follow-up visits in the investigational site prevent us from drawing any definitive conclusions. Taking BIOGUARD-MI and MONITOR-ACS together, the clinical benefit in terms of reducing MACE in post-infarction patients may require longer follow-up or additional therapeutic strategies.

Finally, another interesting finding of our study, in line with previous reports, is that only 30% of symptomatic episodes (activated by the patient) are arrhythmic events in origin (7). Of these, the majority correspond to VPBs, with only 3% of cases corresponding to AF/AFL, NSVT, or ST elevation in a patient complaining of chest pain, later diagnosed as coronary vasospasm. This absence of arrhythmias in symptomatic activations was also observed in the group of patients with “thumb-ECG” intermittent monitoring. This finding is particularly important in clinical practice, as it suggests that many symptomatic episodes post-ACS are not related to arrhythmias.

In summary, this trial emphasizes the clinical utility of ILR in high-risk post-ACS patients, suggesting that its implementation in clinical practice may provide a means for earlier identification of arrhythmias and timely therapeutic intervention, especially in the first months after MI, when most of the clinically relevant arrhythmic events are detected. Nevertheless, the lack of significant reduction in hard clinical outcomes such as death, MI, or stroke indicates that larger studies are needed to define which patients will benefit most from this strategy and definitely establish the impact of post-discharge monitoring on long-term prognosis. We believe that our findings generate valuable hypotheses for the design of future studies with an adequate sample size to assess whether early arrhythmia detection can translate into tangible clinical benefit, particularly in carefully selected high-risk patient populations.

### Limitations

Several limitations of our study should be acknowledged. The main one is clearly the sample size, which prevented us from establishing solid conclusions regarding the prognostic benefits of early diagnosis of post-MI arrhythmias in terms of hard outcomes. Nonetheless, the relevant arrhythmic events detected are surrogates of proven prognostic validity. Regarding the potential obviousness of the findings, however expected the results might be, our results show how many potentially relevant arrhythmic events are often unrecognized in the control group. Frequent symptomatic VPBs were the most frequent type of arrhythmic event recorded by the ILR. Although ventricular ectopy in post-MI patients may be a predictor of heart failure and MACEs, the real impact of this finding in our study is debatable, as current ILR do not provide VPB trends or burden.

Risk stratification in our study was focused on clinical parameters easily available at the bedside, such as the GRACE scale and the CHA_2_DS_2_-VASc. However, a combination of these scores with imaging-based factors might improve risk stratification. Additionally, larger trials should aim for more homogeneous primary endpoints, as combining different types of arrhythmias with varying prognostic relevance may dilute the statistical power to detect meaningful clinical outcomes.

## Conclusion

Post-infarction patients experience a significant burden of arrhythmias. Continuous ECG monitoring with ILR in high-risk ACS patients significantly increased the detection of clinically relevant arrhythmias compared with standard care. This strategy facilitates early therapeutic intervention, potentially modifying the clinical course of these patients, although this was not translated into a difference in 1-year MACE (death, MI, stroke). Future research should focus on identifying which subgroups of post-ACS patients might derive the most benefit from continuous monitoring and whether the early detection of arrhythmias translates into a tangible reduction in adverse clinical outcomes.

## Data Availability

The raw data supporting the conclusions of this article will be made available by the authors, without undue reservation.

## Supplementary material

Supplementary Figure S1 Correlation between symptoms and electrocardiographic findings in the recordings stored by patient activation of the implantable loop recorder.

Supplementary Figure S2 Practical consequences derived from the clinically relevant findings in the monitoring group.

Supplementary Figure S3 Arrhythmia examples from ILR. (1) Atrial flutter. (2) Atrial fibrillation, with recovering of sinus rhythm at the end of the episode. ILR activated by the patient. (3) ILR activation because of angina, with documented runs of NSVT and ST segment elevation. (4) Automatic registry of an NSVT in an asymptomatic patient with incomplete revascularization. (5) VPBs. (6) Complete AV block.
