# Supplementary figures and images for: Evaluation of ambulatory electrocardiographic monitoring of patients after high-risk acute coronary syndrome: the MONITOR ACS-Epic 13 randomized trial
Source: Front Cardiovasc Med. 2025 Aug 18;12:1646175. doi: 10.3389/fcvm.2025.1646175 (PMC12400330; doi:10.3389/fcvm.2025.1646175)

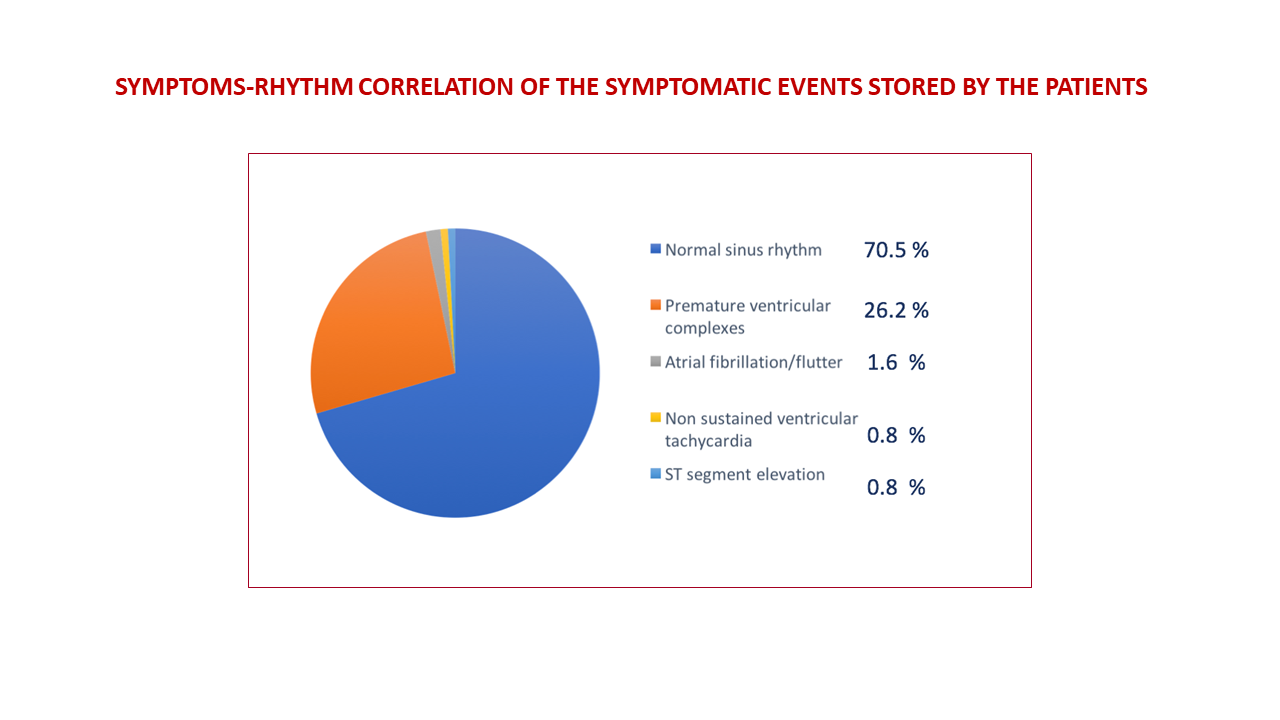

Supplement: Supplementary Figure S1 — Correlation between symptoms and electrocardiographic findings in the recordings stored by patient activation of the implantable loop recorder. [file Image1.tif]

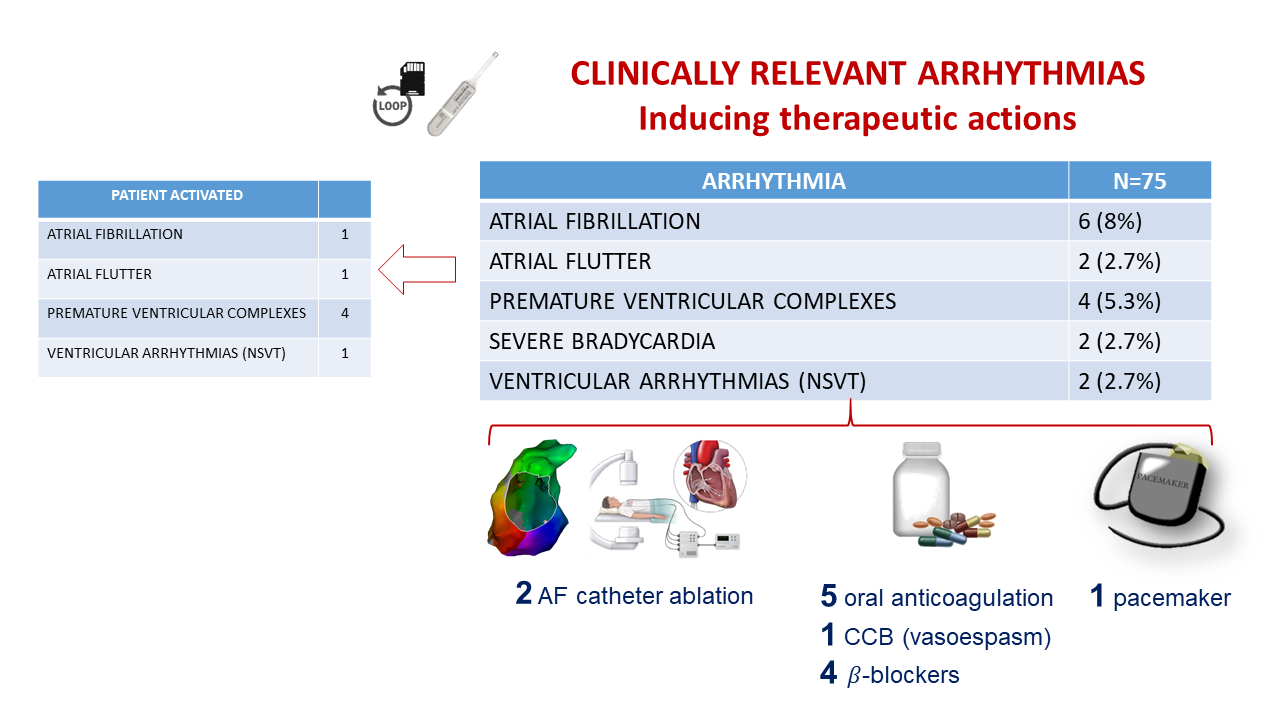

Supplement: Supplementary Figure S2 — Practical consequences derived from the clinically relevant findings in the monitoring group. [file Image2.tif]

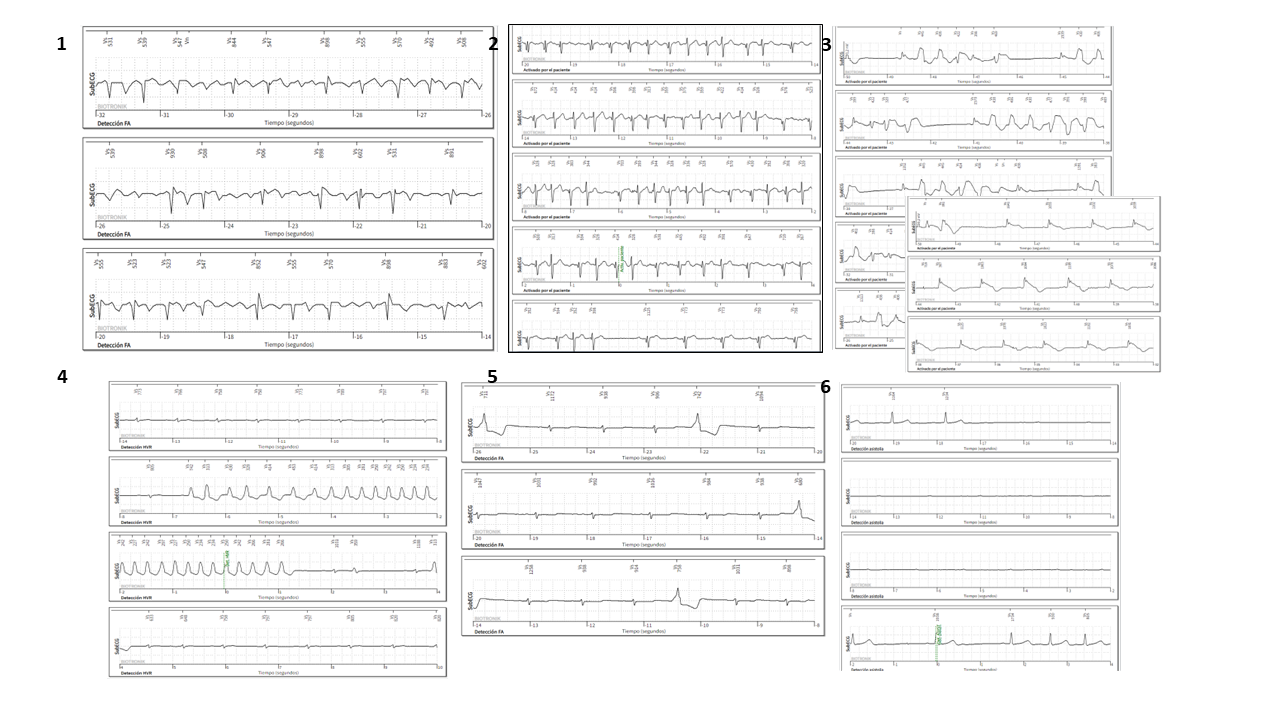

Supplement: Supplementary Figure S3 — Arrhythmia examples from ILR. (1) Atrial flutter. (2) Atrial fibrillation, with recovering of sinus rhythm at the end of the episode. ILR activated by the patient. (3) ILR activation because of angina, with documented runs of NSVT and ST segment elevation. (4) Automatic registry of an NSVT in an asymptomatic patient with incomplete revascularization. (5) VPBs. (6) Complete AV block. [file Image3.png]
