# Supplementary material for: Evaluation of ambulatory electrocardiographic monitoring of patients after high-risk acute coronary syndrome: the MONITOR ACS-Epic 13 randomized trial
Source: Front Cardiovasc Med. 2025 Aug 18;12:1646175. doi: 10.3389/fcvm.2025.1646175 (PMC12400330; doi:10.3389/fcvm.2025.1646175)
Supplement: Supplementary file 4 [file Datasheet1.docx]

**Supplementary Table 1. Findings reported by monitoring with ILR**

**N = 75**

Patients with findings 41 (54.6%)

Patients with clinically relevant findings 16 (21.3%)

Symptoms driven activation 7 / 16 (43.7%)

Patients with non-clinically relevant findings 25 (33.3%)

Symptoms driven activation 3 / 25 (12%)

*Type of findings*

Atrial fibrillation 6 (8%)

Atrial flutter 2 (2.7%)

Atrial tachycardias 3 (4%)

Bradyarrhythmias (AVB, SSS) 12 (16%)

Ventricular tachycardias 2 (2.7%)

Premature ventricular contractions 13 (17.3%)

ST elevation 1 (1.3%)

AVB = atrioventricular block; ILR = implantable loop recorder; SSS = sick sinus syndrome.

**Supplementary Table 2. Analysis for clinical predictors of the primary endpoint**

**Univariant Multivariant**

**HR CI 95% p HR CI 95% p**

Age 0.97 (0.90-1.05) 0.55

Women 0.84 (0.20-3.45) 0.81

Diabetes 1.38 (0.39-4.78) 0.61

Hypertension 1.62 (0.40-6.57) 0.49

Dyslipidemia 0.21 (0.05-0.79) 0.01 0.13 (0.029-0.61) 0.01

Smoker 4.18 (1.04-11.77) 0.02 3.77 (1.01-14.42) 0.04

STEMI 1.14 (0.33-3.90) 0.83

LVEF 1.01 (0.95-1.08) 0.61

GRACE score 1.01 (0.95-1.06) 0.70

CHA₂DS₂-VASc 1.20 (0.65-2.44) 0.42

Total stent length 1.005 (0.98-1.02) 0.60

N treated vessels 1.36 (0.56-3.30) 0.48

LVEF = left ventricular ejection fraction; STEMI = ST elevated myocardial infarction
